# Smoldering Amiodarone‐Induced Decompensated Cirrhosis: Cumulative Dose–Related Toxicity

**DOI:** 10.1155/crgm/9933788

**Published:** 2026-05-24

**Authors:** Jason Ta, Ritesh Bhandari, Joseph Di Francesco, Austin Roberts, Eileen Long, Puneet Chhabra, Junaid Beig

**Affiliations:** ^1^ School of Medicine, University of Tasmania, Hobart, Tasmania, Australia, utas.edu.au; ^2^ Department of Gastroenterology, Royal Hobart Hospital, Hobart, Tasmania, Australia, dhhs.tas.gov.au; ^3^ Department of Anatomical Pathology, Royal Hobart Hospital, Hobart, Tasmania, Australia, dhhs.tas.gov.au

**Keywords:** amiodarone, case report, cirrhosis, hepatotoxicity

## Abstract

Amiodarone is a widely prescribed antiarrhythmic drug, but it can rarely cause severe progressive hepatotoxicity. This report presents a 74‐year‐old man on long‐term amiodarone therapy who developed tense ascites and cholestatic liver dysfunction after 12 years of continuous treatment, corresponding to a cumulative dose of 876 g. Five months prior to presentation, routine liver tests showed subtle and nonspecific abnormalities in liver enzymes, and there were no clinical stigmata of chronic liver disease. Quad‐phase CT imaging demonstrated features of chronic liver disease, with increased hepatic attenuation on the noncontrast phase, consistent with iodine deposition in the context of long‐term amiodarone exposure. Liver biopsy confirmed cirrhosis with ballooning hepatocytes, Mallory–Denk bodies, and neutrophilic satellitosis in the absence of steatosis, histological features suggestive of amiodarone‐induced liver injury. Secondary causes were excluded. Despite discontinuation of amiodarone and initiation of supportive therapy, the patient experienced recurrent decompensation. This case underscores the potential for subclinical progression of amiodarone‐induced liver injury, even in the presence of subtle or nonspecific abnormalities in liver biochemistry without a clear pattern to predict advanced fibrosis or imminent decompensation. Increased hepatic density on noncontrast CT imaging may serve as a radiologic indicator of drug deposition. Long‐term vigilance and monitoring of cumulative dose are essential for early detection and prevention of irreversible liver injury.

## 1. Introduction

Amiodarone, a Class III antiarrhythmic agent, is widely used in the management of atrial and ventricular arrhythmias. Although effective, it is associated with multisystem toxicity, including adverse effects on the lungs, thyroid, eyes, and liver. Hepatic side effects most often present as asymptomatic elevations in liver enzymes. In rare instances, however, amiodarone can cause progressive liver injury, ultimately resulting in cirrhosis and hepatic decompensation.

The hepatic toxicity of amiodarone is attributed to its lipophilic, iodine‐rich structure, leading to hepatic accumulation, phospholipidosis, oxidative stress, and mitochondrial dysfunction [1, 2]. Radiologically, this toxicity may manifest as increased hepatic attenuation on noncontrast CT imaging, serving as an important diagnostic clue due to iodine deposition within hepatocytes [3]. Although other causes of increased liver density exist, such as iron overload or certain metabolic conditions, the pattern seen with amiodarone is typically diffuse and homogeneous, particularly in patients undergoing long‐term therapy.

Histologically, amiodarone‐induced liver injury may mimic alcoholic steatohepatitis, with findings such as ballooning hepatocytes, Mallory–Denk bodies, and neutrophilic satellitosis, typically in the absence of steatosis [4]. Diagnosis relies on clinical history, exclusion of other causes, and histopathological correlation.

This case illustrates amiodarone‐induced cirrhosis diagnosed after clinical decompensation, despite only subtle or nonspecific abnormalities in liver biochemistry and no clear pattern to predict advanced fibrosis or imminent decompensation. It highlights the potential for subclinical progression and the limitations of biochemical monitoring, while emphasizing the supportive roles of imaging and histology in the diagnosis.

## 2. Case Presentation

A 74‐year‐old man presented with tense ascites and cholestatic liver dysfunction. Five months prior to admission, liver tests were largely unremarkable aside from an isolated mild ALT elevation. AST was not measured, and there were no clinical features of chronic liver disease; decompensated cirrhosis was only diagnosed at the time of hospital admission.

His medical history was notable for stable ischemic cardiomyopathy, coronary artery bypass grafting for triple‐vessel coronary artery disease in 2012, hypertension, hypercholesterolemia, and atrial fibrillation. He had been treated with amiodarone continuously from October 2012 to November 2024, corresponding to 12 years of exposure at a daily dose of 200 mg. His other regular medications included warfarin and atorvastatin.

The patient reported only occasional social alcohol consumption within recommended limits and denied the use of over‐the‐counter medications, herbal supplements, or illicit drugs. There was no history of viral hepatitis or a family history of liver disease.

On examination, he was alert and not jaundiced, with no clinical features of hepatic encephalopathy. His abdomen was distended with tense ascites. There were no other peripheral stigmata of chronic liver disease, and no lower‐limb edema.

Liver biochemistry performed in February was within normal limits, while testing in June 2024 showed a mild nonsignificant rise in ALT. In the following months, a progressive cholestatic pattern developed, as evidenced by rising alkaline phosphatase (ALP) and gamma‐glutamyl transferase (GGT) levels, accompanied by declining serum albumin (Table 1). Assessment of the international normalized ratio (INR) and prothrombin time (PT) was confounded by concurrent therapeutic warfarin therapy. Thus, albumin was used as a more reliable marker of hepatic synthetic dysfunction. Serial complete blood counts showed progressive thrombocytopenia, with platelet counts falling from 181 × 10^9^/L to 156 × 10^9^/L over 6 months, consistent with the development of portal hypertension.

**TABLE 1:** Serial laboratory results obtained prior to presentation (June 2024), at presentation (December 2024), and during follow‐up in March and June 2025.

Test	Reference range	June 2024	December 2024	March 2025	June 2025
Hemoglobin (g/L)	130–164	142	140	137	121
Platelets (× 10^9^/L)	160–420	189	181	168	156
WCC (× 10^9^/L)	3.5–9.6	8.7	14.6	12.8	9.2
Neutrophils (× 10^9^/L)	1.8–6.6	5.6	11.6	9.9	7.2
Creatinine (μmol/L)	60–110	94	88	104	87
eGFR (mL/min/1.73 m^2^)	> 90	69	74	70	75
ALP (IU/L)	30–110	102	332	190	159
ALT (IU/L)	5–40	52	16	16	24
AST (IU/L)	5–35	—	32	31	38
GGT (IU/L)	5–50	42	438	257	116
Bilirubin (μmol/L)	< 25	8	14	13	8
Albumin (g/L)	35–52	37	29	34	36
INR^∗^	0.8–1.2	3.0	2.1	2.0	1.9
APTT (s)	27–37	—	—	39	38
PT (s)^∗^	12–16	—	28	26.4	25.2

^∗^Values influenced by concurrent warfarin therapy.

A comprehensive liver screen for secondary causes was negative, including viral hepatitis serology, autoimmune markers (antinuclear antibody, smooth muscle antibody, antimitochondrial antibodies, antisoluble liver antigen, and antiliver–kidney microsomal antibody), alpha‐fetoprotein, immunoglobulin levels, ferritin, ceruloplasmin, serum protein electrophoresis/quantitative serum immunoglobulins, and Alpha‐1 antitrypsin levels.

Ascitic fluid analysis indicated portal hypertension, with a high serum‐ascites albumin gradient (SAAG) of 12 g/L. The fluid was transudative, with low albumin (15 g/L), glucose of 6.2 mmol/L, and LDH of 66 U/L. Cytology was negative for malignant cells, excluding malignancy as a cause of ascites.

**FIGURE 1:**
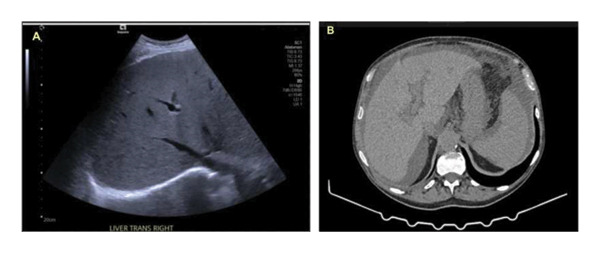
(A) Ultrasound showing increased background echogenicity of the liver. (B) Quad‐phase CT noncontrast liver demonstrating mild increased density of the liver compared with the spleen.

Ultrasound of the liver demonstrated increased background echogenicity, and the two small echogenic lesions in Segments 6 and 8 appeared to be hemangiomas on both scans (Figure 1A). Quad‐phase CT imaging showed signs of chronic liver disease and portal hypertension, such as splenomegaly, peri‐splenic and peri‐esophageal varices, recanalization of the umbilical vein, and moderate ascites. In the noncontrast phase, the mean hepatic attenuation was approximately 70 Hounsfield units (HUs), exceeding the splenic attenuation of 50 HUs, which is consistent with iodine deposition in the setting of chronic amiodarone exposure (Figure 1B).

Transient elastography of the liver showed a fibrosis score of F4 with a median kPa of 75. The scan was technically challenging, rendered difficult by ascites. Nevertheless, the findings were consistent with cirrhosis.

**FIGURE 2:**
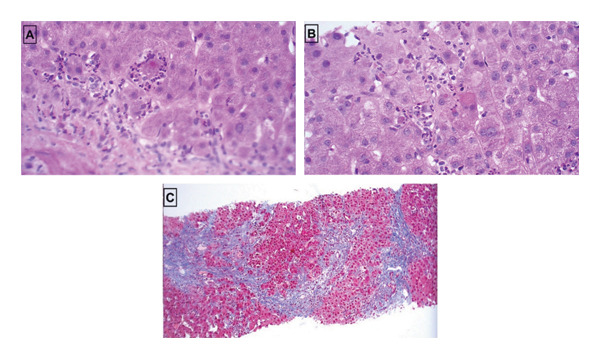
(A) H&E 40x, high power view showing ballooning of hepatocytes with Mallory–Denk bodies. (B) H&E 40x, high power view showing ballooning of hepatocytes, neutrophilic satellitosis, and apoptotic hepatocytes with Mallory–Denk bodies. (C) Masson trichrome, 10x, low power view of nodular architecture with broad bands of fibrous connective tissue surrounding regenerative hepatocyte nodules.

Liver biopsy confirmed established cirrhosis with mixed micronodular and macronodular architecture, broad fibrous septa, ductular proliferation, ballooning hepatocytes, and Mallory–Denk bodies, along with neutrophilic satellitosis (Figure 2A–C). Kupffer cell pigmentation was noted. There was no evidence of steatosis, iron overload, granulomas, or Alpha‐1 antitrypsin inclusions.

Amiodarone was immediately discontinued due to suspected drug‐induced cirrhosis. The patient underwent therapeutic large‐volume paracentesis for tense ascites and was started on spironolactone and furosemide for ongoing ascites management. He was also prescribed carvedilol for primary prophylaxis of variceal bleeding, along with lactulose and subsequently rifaximin for recurrent episodes of hepatic encephalopathy. His gastroscopy showed possible mild portal hypertensive gastropathy without varices.

Nutritional support consisted of a high‐protein diet tailored to patients with cirrhosis. He was enrolled in the hepatocellular carcinoma (HCC) surveillance program and scheduled for regular outpatient follow‐up with hepatology to monitor liver function, cirrhosis complications, and long‐term management.

Despite stopping amiodarone and starting supportive therapy, the patient experienced progressive worsening of liver function. In the months following diagnosis, he required multiple hospital admissions for recurrent tense ascites, spontaneous bacterial peritonitis, and episodes of hepatic encephalopathy, features consistent with ongoing decompensated cirrhosis. Trimethoprim–sulfamethoxazole was commenced for spontaneous bacterial peritonitis prophylaxis. He remains under close hepatology follow‐up and is actively enrolled in a HCC surveillance program. Given the patient’s age and overall clinical context, liver transplantation was not pursued, and management focused on surveillance and supportive care.

**FIGURE 3:**
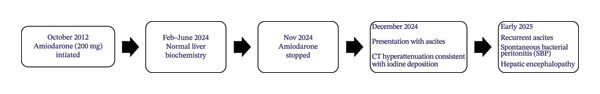
Timeline of long‐term amiodarone exposure, investigations, and clinical course leading to hepatic decompensation.

The chronological sequence of amiodarone exposure, investigations, and subsequent hepatic decompensation is summarized in Figure 3.

## 3. Discussion

Amiodarone, a Class III antiarrhythmic, is widely used to treat atrial and ventricular arrhythmias. While effective in rhythm and rate control, its long‐term use is associated with multisystem toxicity, including pulmonary fibrosis, thyroid dysfunction, ocular complications, and hepatotoxicity. Mild, asymptomatic elevations in aminotransferases occur in approximately one‐quarter of patients, whereas severe hepatotoxicity resulting in cirrhosis is rare but well‐documented [5].

This case highlights a significant diagnostic limitation; advanced amiodarone‐induced liver damage may evolve silently over several years of exposure. The condition frequently manifests as subtle, nonspecific, or fluctuating liver enzyme abnormalities, which may remain undetected until hepatic decompensation develops [5]. Consistent with this limitation, liver tests in our patient 5 months prior to presentation showed only subtle, nonspecific abnormalities and did not suggest advanced chronic liver disease, highlighting that routine biochemistry may provide an incomplete signal of evolving fibrosis.

Amiodarone hepatotoxicity is mediated by the drug’s lipophilic and iodine‐rich structure, resulting in hepatic accumulation of amiodarone and its active metabolite, desethylamiodarone [1]. This leads to phospholipidosis, oxidative stress, mitochondrial dysfunction, and progressive hepatocellular injury [1, 2]. However, while these mechanisms are well established, their clinical expression may be insidious and poorly captured by routine biochemical testing.

Histologically, amiodarone‐induced liver injury often mimics alcoholic steatohepatitis, characterized by ballooning hepatocytes, Mallory–Denk bodies, ductular reaction, and neutrophilic satellitosis [6]. However, a key distinguishing feature is the absence of steatosis, which, in the appropriate clinical context, supports a diagnosis of amiodarone‐induced injury rather than alcohol‐related liver disease [4]. Histological assessment was necessary to establish the diagnosis, reflecting the limitations of biochemical monitoring in detecting advanced fibrosis. Integration of histological, radiological, and clinical findings supported a diagnosis of amiodarone‐induced cirrhosis following exclusion of secondary causes. Histological confirmation was achieved only after clinical decompensation, further underscoring the limitations of enzyme‐based monitoring.

The patient received amiodarone 200 mg daily for 12 years, resulting in a cumulative dose of approximately 876 g. This far exceeds the commonly referenced threshold of 380 g associated with increased hepatotoxicity risk, although such thresholds are derived from observational studies and may not predict individual susceptibility [7]. Case reports indicate variability in latency and doses, with liver injury occurring at markedly lower cumulative exposure [8–11]. Thus, rigid “threshold” values are imprecise and should not be used to reassure clinicians when other clinical features raise concern. In this context, the relatively mild biochemical abnormalities observed months before presentation are more plausibly interpreted as a limitation of enzyme‐based surveillance, rather than evidence of abrupt, late‐stage acceleration. Published experience supports that fibrosis may develop gradually with long‐term exposure and become clinically evident only upon decompensation.

A key radiological observation in this case was increased hepatic attenuation on noncontrast CT imaging, indicative of deposition of radiopaque elements within the hepatocyte, most commonly iodine from amiodarone therapy [3]. Although this finding is nonspecific and may be observed in other conditions, such as iron overload and other storage disorders, a diffuse and homogeneous pattern in a long‐term amiodarone user provides an important contextual clue. This should prompt consideration of drug accumulation and potential chronic hepatotoxicity.

Despite promptly stopping amiodarone, the patient experienced progressive decompensation, with recurrent ascites, spontaneous bacterial peritonitis, and hepatic encephalopathy. This clinical course likely reflects the unique pharmacokinetics of amiodarone, including extensive tissue sequestration and prolonged elimination, resulting in a tissue “depot” effect that may maintain hepatic exposure even after drug withdrawal [12].

Case‐based reviews of amiodarone‐induced cirrhosis report poor short‐term outcomes once cirrhosis is established, with mortality approaching 60% within 5 months, highlighting the importance of earlier recognition and periodic reassessment of ongoing therapy [13].

This case illustrates the central diagnostic limitation that frames the management challenge: periodic liver biochemistry, although widely recommended, failed to signal impending decompensation in our patient. Modest or fluctuating enzyme abnormalities may not reliably reflect the severity or trajectory of underlying fibrosis, and the effectiveness of enzyme‐only monitoring in preventing severe outcomes remains uncertain. The practical implication is that clinicians should not rely on stable or mildly abnormal biochemistry as reassurance against progressive fibrosis in patients on long‐term amiodarone.

This case highlights the need for monitoring strategies that extend beyond routine enzyme testing, especially in patients with prolonged cumulative drug exposure. Noninvasive fibrosis assessment, notably, transient elastography can be incorporated as a monitoring adjunct in analogous drug‐exposure contexts. In methotrexate‐treated cohorts, transient elastography has demonstrated high diagnostic accuracy for detecting advanced fibrosis [14]. In patients with psoriasis and methotrexate‐induced liver fibrosis, there are guidelines that tailor surveillance intervals and the need for liver biopsy with varying transient elastography thresholds [15]. However, these suggestions cannot be directly generalized to patients on amiodarone therapy, and it is critical to acknowledge that amiodarone‐specific thresholds and surveillance protocols have not yet been validated. Caution should be exercised when extrapolating data from methotrexate‐treated cohorts, and such information should primarily be used to generate hypotheses rather than serve as definitive clinical guidance. This case illustrates that reliance on biochemical markers alone is inadequate for fibrosis surveillance. Earlier use of noninvasive fibrosis assessment methods might have detected advanced fibrosis before decompensation.

A pragmatic and cost‐effective approach is therefore to combine routine liver enzyme monitoring with inexpensive adjunctive indicators of evolving advanced liver disease, such as longitudinal trends in platelet count and serum albumin, and, where appropriate, calculation of basic fibrosis triage score such as FIB‐4. Escalation to noninvasive fibrosis assessment (e.g., transient elastography) or hepatology consultation should be considered when indicated. Together, these strategies reflect a shift from enzyme‐based monitoring toward a more integrated assessment of fibrosis risk, directly supporting the central message of this case.

Monitoring the total dose, regularly reassessing the need for ongoing treatment, and considering alternative rhythm‐control strategies are crucial. Future strategies may involve personalized risk assessment, baseline hepatic evaluation before initiating long‐term therapy, and further research into genetic susceptibility. Early recognition and prompt discontinuation of amiodarone are critical to preventing irreversible liver injury.

## 4. Conclusion

Amiodarone‐induced liver injury can progress insidiously to cirrhosis despite only subtle or nonspecific enzyme abnormalities, underscoring the limitations of relying solely on routine liver enzyme monitoring to infer fibrosis trajectory. Diffuse hepatic hyperattenuation on noncontrast CT is a supportive clue to drug deposition in the appropriate clinical context and should prompt targeted evaluation for chronic liver injury. Histological features of amiodarone‐induced liver injury may mimic alcoholic steatohepatitis, typically without steatosis. Awareness of cumulative dose and diligent long‐term surveillance are essential for early detection and prevention of irreversible liver damage in patients receiving chronic amiodarone therapy.

## Funding

No funding was provided for this case report.

Open access publishing facilitated by University of Tasmania, as part of the Wiley ‐ University of Tasmania agreement via the Council of Australasian University Librarians.

## Consent

Written informed consent was obtained from the patient for the publication of this case report. A copy of the written consent form is readily available to be provided to the journal’s editorial office or ethics committee on request.

Written informed consent was obtained, with a signed consent form readily providable at request.

## Conflicts of Interest

The authors declare no conflicts of interest.

## Data Availability

The data that support the findings of this study are available from the corresponding author upon reasonable request.
